# Rediscovery of *Bembidion (Lymnaeum) nigropiceum* (Marsham) (= *puritanum* Hayward) in Massachusetts, with remarks on biology and habitat (Coleoptera, Carabidae, Bembidiini)

**DOI:** 10.3897/zookeys.147.2105

**Published:** 2011-11-16

**Authors:** Robert L. Davidson, Jessica Rykken

**Affiliations:** 1 Section of Invertebrate Zoology, Carnegie Museum of Natural History, 4400 Forbes Avenue, Pittsburgh PA 15213; 2 Museum of Comparative Zoology, Harvard University, 26 Oxford Street, Cambridge, MA 02138

**Keywords:** Boston Harbor Islands, introduced established species, rediscovery, intertidal habitat

## Abstract

*Bembidion (Lymnaeum) nigropiceum* (Marsham) (=*puritanum* Hayward), a European species introduced into Massachusetts but presumed not to have become established, has been rediscovered during the Boston Harbor Islands All Taxa Biodiversity Inventory undertaken by the Museum of Comparative Zoology and the National Park Service. A summary is presented of treatment of this species in North America. Data on specimens collected are presented, along with observations on habitat and biology. Some speculations are presented about its highly specialized habitat in the gravel pushed up by high tide, which may act as a food-trapping sieve. A few words are included about future actions needed to resolve questions of distribution and behavior.

## Introduction

For young carabidologists growing up in New England, *Bembidion puritanum* Hayward was something of a holy grail. We all knew about it, knew it looked weird for a *Bembidion*, knew it was known from a few specimens labelled “Massachusetts” and never found again, knew nothing was known about the habitat, and knew that we would give our eye teeth to be the first to find the thing again. Surprisingly, Lindroth (1963) himself did not realize it was actually an introduced species, so it remained in his great monograph as a tantalizing prize for which to be searched. Eventually it was recognized as an adventive species (Erwin and Kavanaugh 1980), and the wonderful name “puritanum” fell as a synonym to the more prosaic “nigropiceum.” This was unfortunate as the name “puritanum” would now have been doubly appropriate, both for its presence in Massachusetts and for its pilgrimage across the Atlantic to get there. In 1980 no new specimen had been seen for 83 years, the species was therefore presumed to have been introduced but not established, and this seemed to lay the matter to rest. Until now. To our utter astonishment (and one of the most thrilling carabid moments of our lives), in doing routine identifications on survey material from the Boston Harbor Islands All Taxa Biodiversity Inventory (ATBI), we found four specimens of this elusive species (and later collected many more). As if fate had intervened, the authors had converged from Pittsburgh and Boston on a lab at the University of Vermont to do this work, and thus our esteemed mentor and friend Ross T. Bell happened to be present at the eureka moment. It is to Ross and Joyce Bell that we dedicate this paper, in recognition of many decades of learning, fieldwork, and sheer fun.

## History

**Figures 1–2.**
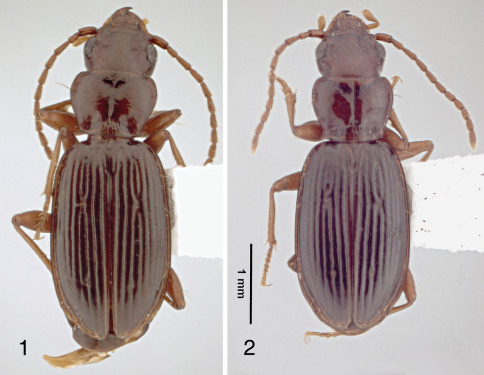
Dorsal habitus of male *Bembidion nigropiceum* collected on Thompson Island, May 7, 2008. Automontage images by J. Rawlins.

Hayward (1897) described *Bembidion puritanum* from four specimens from Massachusetts. There was no further indication of locality and no information on habitat. The species is unusual among North American *Bembidion* (until 1980 it was presumed to be a North American species), so it provoked more than average interest. It has small eyes, short wings, a brown color, and a not very *Bembidion*-like gestalt (Figs 1–2). It is superficially similar to other oddities like *Amerizus*, *Cillenus*, and a couple of other species of *Bembidion* (*Lymnaeum*) (about which more below). All this stimulated many collectors for many years to search for it, but no further specimens were ever turned up.

Lindroth himself, uncharacteristically, did not recognize that this was, in fact, a European species. This is puzzling, as Lindroth was a *Bembidion* specialist, but the species does not occur in Scandinavia, where Lindroth cut his carabid teeth, is relatively rare (in collections) with very spotty distribution even in Europe, and does not occur in Canada or Alaska where Lindroth’s attention was focused. So perhaps he was unfamiliar with it until later in life (he certainly knew it by the time he worked on the carabid fauna of England (Lindroth 1974)); or perhaps he just assumed it was a similar but different species. Whatever the reason, it remained *Bembidion puritanum* in Lindroth’s monumental monograph (1963 for *Bembidion*) on the Carabidae of Canada and Alaska, with a special place at the very end of more than two hundred pages of North American *Bembidion*.

Eighty-three years after its description, at last the penny tumbled. Erwin and Kavanaugh (1980), while researching for the description of a California species, realized that *Bembidion puritanum* Hayward, 1897, and *Bembidion nigropiceum* (Marsham, 1802) were synonyms, and that the American species was actually introduced from Europe. Their conclusion was that the species was probably introduced in ship’s ballast in a Massachusetts harbor in the 1800’s and lasted at least one generation, as one of Hayward’s specimens is teneral; but they doubted the species was still resident in North America. They continue “[if] *Bembidion nigropiceum* became established and still survives in North America, we suggest it be looked for in natural areas adjacent to harbors that received ships in the 1800’s.” For another thirty years, this was the end of the matter, and the only subsequent mentions refer to it as “introduced, probably not established” (Bousquet and Larochelle 1993) or “doubtfully established in North America” (Larochelle and Larivière 2003). But the remarks of Erwin and Kavanaugh proved to be amazingly perspicacious.

In October, 2007, one hundred ten years on from Hayward’s description, Davidson and Rykken converged on Burlington, Vermont, to meet in Ross Bell’s lab to identify the latest round of carabids from the Boston Harbor Islands ATBI. This project is a six-year collaborative effort between the MCZ and the National Park Service to catalogue arthropod diversity in Boston Harbor Islands national park area. There had been some interesting material up to that point, but nothing compared with the excitement that ensued when the first specimen of *Bembidion nigropiceum* turned up, as mentioned in the introduction. The eventual total was four specimens from Thompson Island from June 20th through July 13th, with the July specimen being teneral. The excitement gradually subsided, and we began thinking about how to follow up on this discovery. Assuming a July teneral might imply some kind of adult activity roughly two or three months prior (enough time for egg-laying, larval development, and pupation), we calculated late April to early May would be the best time to mount a careful search near the pitfall site. On May 7, 2008, we proceeded to the site. The traps were high on the beach, but we knew from Lindroth (1974), Jeannel (1941) and others (see Neri and Magrini 2010 for a full list of citations) that in Europe the beetles were thought to be confined to the tidal zone, so we made a beeline for the gravel beach. Success was immediate. In the mound of pushed-up gravel between the high tide line and the dry upper beach, our beetle was abundant. We collected more than 60 specimens along this “rind” of gravel for a couple of hundred yards along the beach, none above or below this line; and subsequent hand-collecting and pitfalling produced a few more specimens on this island and two others. It was clear *Bembidion nigropiceum* was alive and well in North America, and probably had been here all along.

We realize that it cannot be proven that these populations are descended from the same introduction event(s) that produced Hayward’s specimens, and not from subsequent introduction; but it is the most likely scenario. Given their elusive nature even in their homelands; their very specialized habitat and narrow habitat zone; their tiny size, winglessness and slow dispersal rate; and the paucity of collectors, it is likely they have been here all along, introduced sometime probably well before 1897.

The species is closely related to two other species in the Mediterranean, and perhaps more distantly to two species in the American west (California and Utah). Serendipitously, in late 2010, when we were already set in our thoughts about habitat and behavior of *Bembidion nigropiceum* in the Boston Harbor Islands, an excellent paper by Neri and Magrini was published. It deals exhaustively with matters taxonomic and distributional for all three European species of subgenus *Lymnaeum*, and it has an excellent summary of the rather sparse literature mentions of the habitat of *Bembidion nigropiceum* in Europe. An embryonic version of the discoveries presented here was given in the Vermont Entomological Society Newsletter (Rykken and Davidson 2008).

## Methods

See Davidson et al. (2011) for an overall discussion of the Boston Harbor Islands All Taxa Biodiversity Inventory; the islands; results and analysis for Carabidae as a whole; sampling design and protocols; comparison with the mainland fauna and discussion of other introduced species. For more information about the Boston Harbor Islands ATBI (including a database with images and a summary of research on the distribution of carabid species across the islands), go to: http://insects.oeb.harvard.edu/boston_islands

The following acronyms are used:

CMNH Carnegie Museum of Natural History, Pittsburgh, Pennsylvania USA.

CNC Canadian National Collection, Ottawa, Ontario, Canada.

DMC David Maddison Collection, currently at OSU.

MCZ Museum of Comparative Zoology, Harvard University, Cambridge, Massachusetts USA.

OSU Oregon State University, Corvallis, Oregon USA.

## Results

A total of 82 specimens was collected as follows:

Massachusetts. Suffolk County. Grape Island, 12-25 June 2008, pitfall (one female). Rainsford Island, 15 July 2008, hand-collected (four males, five females). Thompson Island, 20-26 June 2007, pitfall (two males, one female); 3-13 July 2007, pitfall (one male); 7 May 2008, hand-collected (24 males, 37 females); 19 June 2008, hand-collected (one male); 9 July 2008, hand-collected (two males, three females); 28 May 2010, hand-collected (one specimen, gender not checked).

These, along with the original type series at MCZ (4 specimens), are all known specimens of *Bembidion nigropiceum* collected in North America to date, a total of 86 individuals. All specimens are at MCZ or CMNH, except pairs given to Yves Bousquet at the CNC and David Maddison (DMC) at OSU. Gender was noted for 81 of the specimens, and there are 34 males (42%) and 47 females (58%). There is not yet sufficient material from collections later in the year to make an informative comparison with sex ratios in the early spring (May 7, 2008: 24 males (39%) and 37 females (61%)). Of the 62 specimens collected in May, not one was teneral. Of the twenty specimens collected in late June and into July, twelve were teneral (60%).

### Observations and Discussion

**Figure 3.**
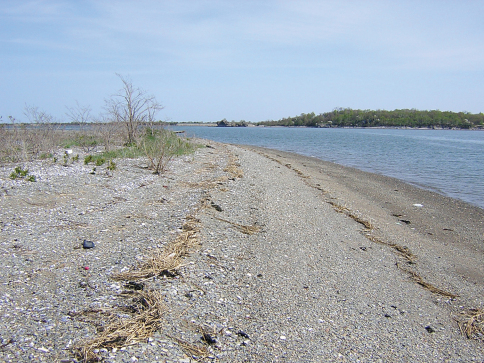
Photograph of the gravel “tube” at high-tide line on Thompson Island, looking away from the city of Boston. Photo by R. E. Acciavatti.

**Figure 4.**
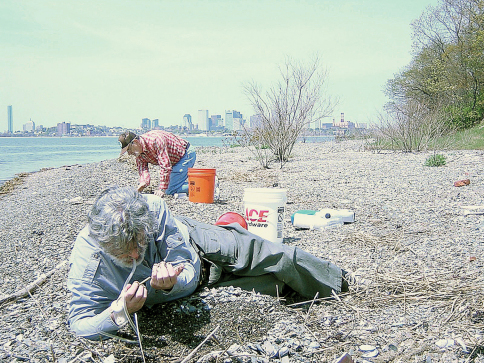
Closer view of the gravel “tube” on Thompson Island, looking toward the skyline of Boston, “tube” partly excavated in foreground. Collectors are Robert L. Davidson (foreground) and Robert E. Acciavatti (background). Photo by J. Rykken.

**Figure 5.**
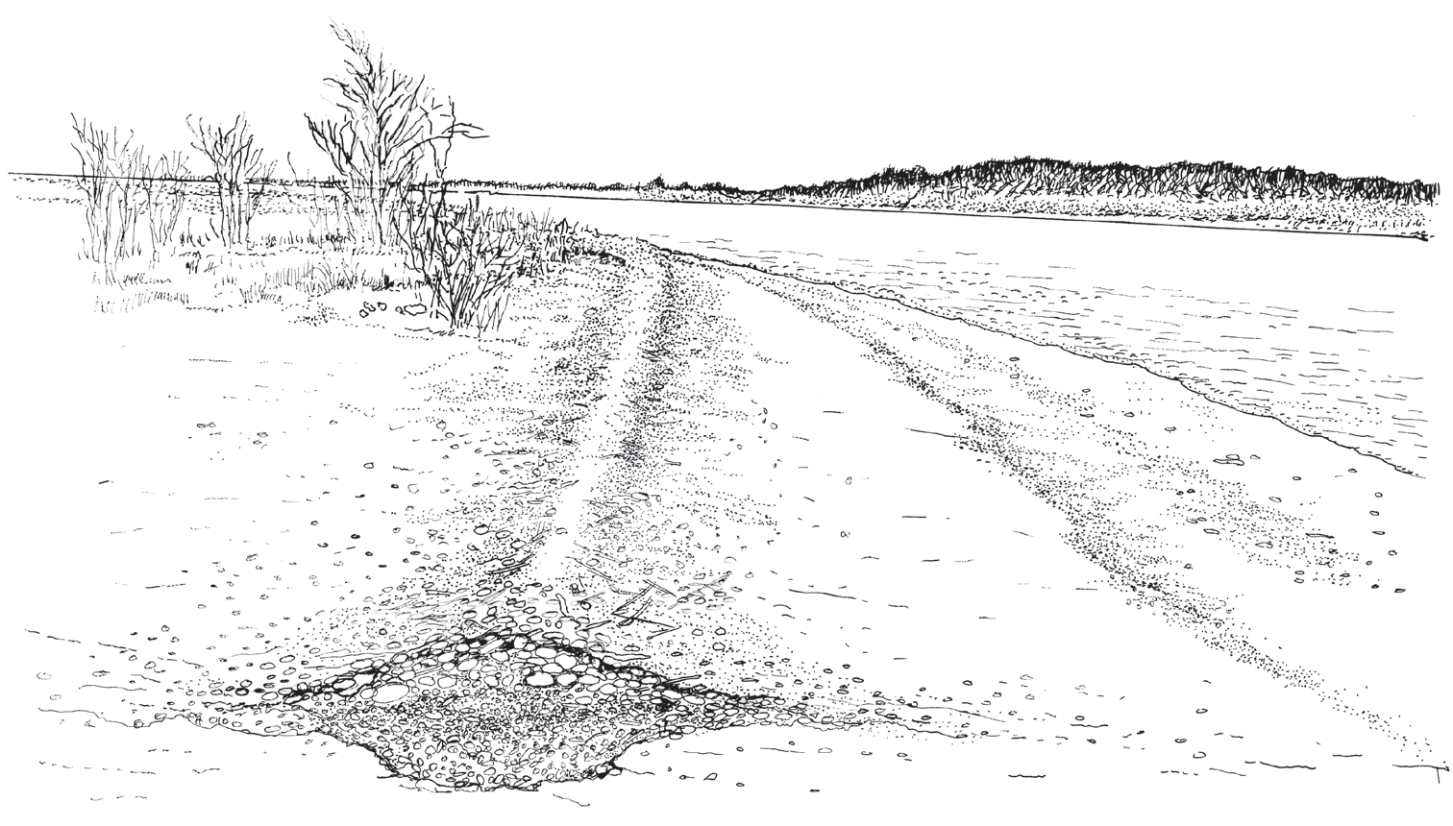
Cross-sectional drawing of beach at Thompson Island site showing mound of gravel and underlying depression created by seawater at high tide. Seawater is to the right, high ground and vegetation is out of sight to the left. Distance across the widest part of the gravel “tube” is about a meter. Drawing by J. Hyland.

Figures 3 and 4 show images of the gravel beach on Thompson Island at which most of the specimens were taken, including the “rind” or “nirdle” of gravel pushed up by the seawater at the high-tide line. Figure 5 is a cross-sectional drawing of the beach and the “nirdle.” The latter is essentially a tube of gravel from which most of the sand has been leeched by the seawater percolating through it at high tide. In cross-section, the tube is roughly a meter wide from water-side to land-side, perhaps half to two-thirds of a meter from top to bottom (about half of this above the hard sand bottom and half gouged into the sand bottom), and runs the entire length of the gravel beach. On May 7, 2008, when the longest series was hand-collected, ALL specimens were taken inside this tube of gravel. Between water line and this mound, and on the drier beach shoreward from this mound, and even on the dry top and sandy bottom of this mound, few (if any) beetles were found. But in the core of this mound, in the more or less sandless interstices of the very wet gravel, beetles were abundant and were moving actively about, presumably foraging for something. This is somewhat speculative, but it was our impression that the gravel mound acted as a massive sieve, where the lapping seawater at high tide was strained through the gravel (thus leeching it of sand). It thereby mimicked the baleen plates of a whale, trapping small sea organisms, some of them presumably soft and tasty morsels that might provide sustenance for the beetles. The gravel is relatively coarse, but not cobble-sized, varying from pea-size to ping pong ball, on average smaller grained toward the top.

We do not know whether at this early season all beetles remain in this zone, whether they migrate inland during bad weather, nor whether they remain in this zone at night. We do know that later in the season at least some individuals can be found higher on the beach (the original series from 2007 was taken in late June and early July high on the beach not far from the beginnings of the vegetated zone). The species is short-winged and no full-winged individual has yet been reported, so moving some distance is no trivial matter; movement inland presumably has a purpose. We do not know whether at some periods they remain in the upper beach zone or are merely in transit to much higher ground. We did not observe any individuals *in copula*, so we do not know whether mating, egg-laying, or larval development takes place in the gravel mound, on higher ground, or elsewhere. Three of the five specimens collected in late June and July in pitfalls higher on the beach were teneral (60%), which suggests pupation higher on the beach, or pupation on even higher ground followed by migration back to the beach. It is not certain where on the beach the remaining June-July specimens were taken. In total, though, there were twenty specimens taken in June-July, of which twelve were teneral (again 60%); 62 specimens were taken in May, and not one was teneral. So it seems certain that egg-laying, larval development and pupation occur in May and June with hatching in late June and into July. There is at least some adult activity in the gravel mound throughout this time (hand-collected specimens of June 19 and July 9 from Thompson Island and July 15 from Rainsford Island), but we do not know how it compares with the spring activity, or whether there is a second generation of adult activity in this zone in the fall, or no new activity until the following spring. Clearly there is a great opportunity for study here to track the life history of this species through an entire season.

We should mention that on May 7, 2008, no other carabids were found mixed with *Bembidion nigropiceum* except one: *Apristus subsulcatus* (Dejean) outnumbered the former about two to one (there were a few *Apristus latens* (LeConte), but as we did not distinguish these in the field, we do not know whether they occurred in the gravel mound or elsewhere on the beach). *Apristus subsulcatus* occurred along with *Bembidion nigropiceum* in the gravel mound, but it was not confined to it. The former was more or less uniformly abundant in the gravel on the higher beach, and less abundant but still present in gravel seaward of the mound and thus twice a day under water. These beetles are good fliers and can presumably get out of the way of incoming tide.

*Bembidion nigropiceum* does not fly so far as known; no winged individual has yet been reported. It is not impossible that an occasional winged individual is produced for dispersal, but it seems unlikely as in such cases there are usually some young colonies with a few winged individuals in the first few generations, and none such has been found for *Bembidion nigropiceum*. Its small eyes also suggest it is not a flier. It should be noted, however, that the closely related *Bembidion abeillei* Bedel is wing-dimorphic. *Bembidion nigropiceum* presumably disperses primarily in drift material.

The earliest reference to habitat for *Bembidion nigropiceum* (see Neri and Magrini 2010 for a thorough review) is Chaudoir (1846), who describes the habitat as under vegetation thrown up by the sea (“sous les herbes rejetées par la mer”). Thereafter, a number of authors mention that it seems to be limited to gravel and shingle near the high-tide line, and Jeannel (1941) states that the beetles remain under water at high tide (“se laissant recouvrir par la mer à marée haute”), the latter confirmed by several other observers. No one has yet commented on the possible sieve-like action of the gravel and tide in straining small organisms. The habitat observation closest to ours is by Gridelli (1944), which translates as: undoubtedly linked to the pebble beach; it can be found by digging in the gravel until you reach an underlying layer of coarse sand infiltrated by the seawater.

What about the future? It appears that Boston Harbor has been the introduction point for *Bembidion nigropiceum*, and is possibly still the only place in North America in which *Bembidion nigropiceum* occurs. It has been found on three islands already and will undoubtedly turn up on others where there is suitable habitat. It will be surprising, too, if it is not already on the mainland somewhere in Boston Harbor, if there is suitable habitat, and searching the harbor mainland would be a logical first step in trying to find how far this species may have dispersed. The species probably disperses primarily in drift, so it should be searched for diligently to the northeast of Boston Harbor, where prevailing currents and winds are most likely to have taken it. There is plenty of suitable gravel-beach habitat along the mainland coast through the rest of Massachusetts, New Hampshire and Maine, and it is in this direction that it is more likely to have dispersed already. Small size, extremely narrow habitat zone, paucity of collectors, and difficulty of recognition may have prevented discovery so far, but perhaps this report and its images will help remedy that. It will be even more interesting to find whether it has been able to break out of Boston Harbor to the south and southwest. This might have been more difficult, as the prevailing currents off Cape Cod head northeast, and the sandy habitats of Cape Cod to the east and south of Boston Harbor might be difficult for this beetle to get across. But there is plenty of good habitat along the coasts of Rhode Island, Connecticut and Long Island that would be well worth investigating. We hope this fine beetle is thriving in North America, and that we will be hearing from someone soon.
